# Longitudinal prediction of BMI using explainable AI: integrating polygenic scores, maternal, early-life and familial factors

**DOI:** 10.1038/s41366-026-02050-1

**Published:** 2026-03-16

**Authors:** Fuling Chen, Phillip E. Melton, Kevin Vinsen, Trevor Mori, Lawrence Beilin, Rae-Chi Huang

**Affiliations:** 1 International Centre for Radio Astronomy Research, University of Western Australia, Perth, WA Australia; 2 Menzies Institute for Medical Research, University of Tasmania, Hobart, TAS Australia; 3 Medical School, University of Western Australia, Perth, WA Australia; 4 Nutrition & Health Innovation Research Institute, Edith Cowan University, Perth, WA Australia

**Keywords:** Obesity, Development

## Abstract

**Background/objectives:**

This study aimed to predict body mass index (BMI) trajectories from childhood to early adulthood using explainable artificial intelligence, integrating adult BMI polygenic scores (PGS), maternal, early-life, and familial factors to identify key predictors of obesity risk and inform prevention strategies.

**Subjects/methods:**

We analyzed longitudinal data from the Raine Study Gen2 cohort, recruiting 2868 participants. This observational study, without randomization or case-control design, collected BMI measurements at ages 8, 10, 14, 17, 20, 23, and 27 years. We applied Kolmogorov–Arnold Networks (KAN) alongside conventional machine learning models, integrating epidemiological variables (maternal and paternal anthropometrics, parental education, early-life skinfold measurements) with seven BMI-related PGS. The analysis spanned from childhood to early adulthood, with no intervention administered.

**Results:**

The KAN model, combining epidemiological and PGS data, achieved predictive performance with R² ranging from 0.81 for BMI at age 8 to 0.34 at age 27. BMI z-score at age 5 was the dominant predictor in early years, with adult BMI PGS influence increasing post-adolescence. Maternal and paternal anthropometry, parental education, and early-life skinfold measurements were significant contributors.

**Conclusions:**

The interpretable KAN model revealed the dynamic interplay of childhood BMI z-score and PGS emerging as key drivers of BMI trajectories across life stages. The finding underscores the potential of BMI at critical time in early childhood as a biomarker for obesity risk. Our interpretable model offers actionable insights for targeted obesity prevention strategies.

## Introduction

Obesity, defined as an excessive accumulation of body fat that poses health risks, has reached epidemic proportions globally. Worldwide, adult obesity has more than doubled since 1990, and adolescent obesity has quadrupled (WHO) [1]. In Australia, nearly two-thirds of adults and one in four children are overweight or obesity, underscoring the need for targeted interventions and prevention. Childhood obesity often persists into adulthood, with longitudinal studies showing that ~55% of children with obesity remain with obesity in adolescence, 80% of adolescents with obesity continue as adults, and 70% maintain obesity beyond age 30 [2]. This highlights the long-term implications and importance of early prevention.

Key factors influencing obesity development from childhood to early adulthood are complex and multifaceted, including environmental, biological, pre- and postnatal, and psychosocial factors [3]. Children of parents with obesity face higher risks [4], with stronger associations in older children, reflecting genetic, epigenetic, and shared environmental influences [4]. Economic factors affect physical activity levels and access to healthy food [5]. Pre- and postnatal factors such as maternal pre-pregnancy obesity [6], excessive gestational weight gain, gestational diabetes [6, 7], and smoking [8] are significant contributors. Early childhood factors, including birth weight, rapid postnatal weight gain [9, 10], and breastfeeding duration [11], have also been linked to obesity risk.

Body mass index (BMI) serves as a surrogate measure of obesity risk at the population level [12]. Our study is motivated by the need to deepen our understanding of the longitudinal impact of a range of factors identifiable early in life, that influence BMI development into adulthood, incorporating insights from early childhood, parental, environmental and genetic factors. Unlike previous studies, which often focus on isolated factors or lack comprehensive interpretability, we proposed a novel machine learning (ML) model that balances predictive performance with robust interpretability. Additionally, we introduced an innovative methodology to systematically identify and quantify the importance of influential factors, transforming these insights into precise BMI estimations.

## Materials and methods

### Study population

This study used data from the Raine Study Gen2^1^ (the variables in the Rain Study are mostly harmonized with the LifeCycle Project-EU Child Cohort Network [13]). The Raine Study is a large, well-established longitudinal cohort designed to track health and developmental outcomes from pregnancy into adulthood [14]. The Raine Study initially recruited 2900 pregnant women (Generation 1, Gen1) and followed 2868 children (Generation 2, Gen2). The Gen2 cohort has been followed up longitudinally from birth into early adulthood [14]. Table 1 summarizes the key characteristics, including environmental, maternal, parental and early childhood factors across seven age groups (8–27 years) in the Raine Study.

**Table 1:** Descriptive statistics and significance of key characteristics across ages 8–27 years in the Raine Study.

Variables	8 yrs	10 yrs	14 yrs	17 yrs	20 yrs	23 yrs	27 yrs
Sample size	*N* = 1046	*N* = 1001	*N* = 986	*N* = 810	*N* = 807	*N* = 584	*N* = 645
BMI (kg/m^2^)							
Mean (std)	16.91 (2.50)	18.73 (3.39)	21.41 (4.15)	22.92 (4.28)	24.48 (4.93)	25.18 (5.06)	25.81 (5.54)
Range	(12.28, 31.31)	(13.14, 36.80)	(14.12, 43.79)	(15.65, 44.31)	(15.97, 48.89)	(16.50, 46.74)	(17.42, 62.13)
Environmental factors							
Household income^a^		*	***	**	***	*	*****
4th quartile (highest)	40%	41%	40%	43%	42%	44%	42%
3rd quartile	27%	27%	27%	25%	27%	26%	28%
2nd quartile	21%	21%	22%	21%	20%	19%	20%
1st quartile	12%	11%	11%	11%	11%	11%	10%
Father’s education^b^			*		***		***
High	51%	52%	50%	52%	53%	54%	55%
Medium	18%	18%	18%	18%	19%	21%	19%
Low	31%	30%	32%	30%	27%	25%	26%
Maternity factors							
Smoking before pregnancy	*	***	****				
No	45%	46%	45%	47%	47%	50%	47%
Yes	55%	54%	55%	53%	53%	50%	53%
Pre-pregnancy weight (kg)	#	#	#	#	#	#	#
Mean (std)	60.33 (11.63)	60.40 (11.67)	60.54 (11.64),	60.07 (11.54)	60.22 (11.66)	60.31 (11.60)	59.80 (11.09)
Range	(35.00, 129.00)	(35.00, 129.00)	(35.00, 129.00)	(35.00, 129.00)	(35.00, 129.00)	(35.00, 125.00)	(35.00, 129.00)
Parental factors							
Mother’s BMI (kg/m^2^)	#	#	#	#	#	#	#
Mean (std)	22.39 (4.08)	22.42 (4.11)	22.41 (4.10)	22.26 (4.08)	22.34 (4.11)	22.33 (4.08)	22.14 (3.93)
Range	(13.06, 48.83)	(13.06, 48.83)	(13.06, 48.83)	(13.06, 48.83)	(13.06, 48.83)	(13.06, 48.83)	(13.06, 48.83)
Father’s BMI (kg/m^2^)	#	#	#	#	#	#	#
Mean (std)	24.52 (3.26)	24.56 (3.29)	24.60 (3.36)	24.52 (3.31)	24.47 (3.25)	24.54 (3.19)	24.39 (3.14)
Range	(16.48, 41.52)	(16.12, 41.52)	(16.12, 41.52)	(16.12, 41.52)	(16.12, 38.58)	(16.12, 38.58)	(16.12, 36.33)
Mother’s age at birth (yr)							
Mean (std)	28.98 (5.71)	29.16 (5.67)	29.01 (5.76)	29.29 (5.73)	29.27 (5.75)	29.74 (5.63)	29.69 (5.74)
Range	(15.00, 46.00)	(15.00, 46.00)	(15.00, 43.00)	(15.00, 43.00)	(15.00, 43.00)	(16.00, 43.00)	(16.00, 46.00)
Father’s age at birth (yr)							
Mean (std)	31.27 (6.65)	31.35 (6.62)	31.28 (6.76)	31.61 (6.90)	31.47 (6.74)	31.89 (6.66)	31.76 (6.73)
Range	(15.00, 59.00)	(15.00, 59.00)	(15.00, 59.00)	(15.00, 59.00)	(15.00, 59.00)	(18.00, 58.00)	(17.00, 58.00)
Early childhood factors							
Sex			*				
Male	52%	53%	53%	52%	53%	53%	51%
Female	48%	47%	47%	48%	47%	47%	49%
Weight at birth (g)							
Mean (std)	3377.05 (545.63)	3384.21 (548.75)	3378.69 (551.57)	3371.62 (536.40)	3378.02 (528.50)	3394.08 (514.39)	3398.23 (544.09)
Range	(760.00, 5550.00)	(750.00, 5550.00)	(750.00, 5550.00)	(1020.00, 5550.00)	(1020.00, 5185.00)	(1020.00, 5185.00)	(1020.00, 5550.00)
Length at birth (cm)							
Mean (std)	49.15 (2.36)	49.15 (2.43)	49.15 (2.46)	49.18 (2.29)	49.19 (2.33)	49.25 (2.26)	49.31 (2.33)
Range	(34.50, 57.00)	(30.50, 57.00)	(30.50, 57.00)	(39.00, 57.00)	(39.00, 57.00)	(39.00, 56.00)	(39.00, 56.00)

^a^Total yearly income of the household categorized into quartiles of low, medium-low, medium-high and high income levels based on the national yearly household income distribution.

^b^Education level categorized into High (Short cycle tertiary, Bachelor, Masters, Doctoral or equivalent), Medium (Upper secondary, Post-secondary non-tertiary), and Low (No education; early childhood; pre-primary; primary; lower secondary or second stage of basic education).

*p < 0.05; **p < 0.01; ***p < 0.005; ****p < 0.001.

#|*r*| > 0.2; ##|*r*| > 0.4; ###|*r*| > 0.6; ####|*r*| > 0.8.

### Outcome measure

Gen2 underwent phenotyping for BMI at ages 8, 10, 14, 17, 20, 23, and 27. Weight was measured using standardized scales, and height was recorded using a stadiometer. BMI was calculated as weight (kg) divided by height squared (m²) and served as the primary outcome variable. Descriptive statistics for BMI across these age groups are presented in Supplementary 1.

### Epidemiological predictors – maternal, early childhood and family variables - *(Epidemiology Dataset)*

The *Epidemiology Dataset* comprises 201 raw variables (see descriptions and statistics in Supplementary 1) grouped into four categories: environmental factors (paternal characteristics, household socioeconomic conditions, family structure and environmental exposures), maternal factors (pregnancy health, anthropometrics and lifestyle), other parental factors and early-life anthropometrics (neonatal and early childhood anthropometrics at birth, 1 and 5 years). To ensure robust analysis, we cleaned the data and applied correlation-based clustering to group similar variables, then selected the most important predictors for BMI. Participants with missing data were removed to create an optimized dataset. See Supplementary 1 for variable descriptions and statistics, and Supplementary 2 for data flow and sample sizes.

### Genetic factors - (Genetic Dataset)

Seven polygenic scores (PGS) for BMI (*PGS002313* [15]*, PGS002161* [16]*, PGS00027* [17]*, PGS004150* [18]*, PGS003884* [19]*, PGS002853* [20]*, PGS000921* [21]) were sourced from the PGS Catalog [22]. Each score reflects a genetic predisposition to BMI, based on genetic variants identified in large-scale genome-wide association studies (GWAS). The seven PGSs were chosen for their large number of variants (over one million) and diverse development methods as listed in Table 2.

**Table 2:** The summary of the PGS used in this paper.

PGS ID	PGS publication ID	Number of variants	Development method	Score development		
				Sample number	Sample ancestry	Cohort
PGS002313	PGP000332	1,109,311	BOLT-LMM	336,394	European	UKB
PGS002161	PGP000263	990,022	LDpred2 (bigsnpr)	391,124	European	UKB
PGS000027	PGP000017	2,100,302	LDpred	119,951	European	UKB
PGS004150	PGP000517	1,039,042	UKBB-EUR.MultiPRS.CV	359,913	European	UKB
PGS003884	PGP000501	1,067,771	PRS-CSx	49,335	Hispanic or Latin American, African American or Afro-Caribbean, East Asian, Asian unspecified, Oceanian, Native American	NR
PGS002853	PGP000393	7,446,664	DBSLMM	23,381	European	MGI
PGS000921	PGP000243	1,947,711	LDpred	1000	European (Danish)	Inter99

*NR* Not Reported.

The PGS were calculated using the pgsc_calc pipeline [23], which computes PGS by combining genetic data with scoring files from the PGS Catalog. The pipeline first lifts variant coordinates from their original genome build to the target build (GRCh38), then matches genetic variants (SNPs) from the target dataset to those in the PGS Catalog scoring file, using chromosome, position, and allele information. After removing the problematic variants, such as those with missing or unclear data, the pipeline multiplies the number of effect alleles (0, 1, or 2) by their effect weights (from GWAS) for each matched SNP and adds them up to get the PGS for each sample, which indicates an individual’s genetic risk for higher BMI. The pipeline finally adjusts the scores to make them comparable across individuals.

After preprocessing and clustering the *Epidemiology* and *Genetic Datasets* to address data multicollinearity, we used Recursive Feature Elimination method to select the top predictors that achieved the highest scores. These predictors were subsequently used to train the model and analyze the outcomes.

### Machine learning models

This study utilized four conventional machine learning models: Extreme Random Forest [24] (ERF), Extreme Gradient Boosting [25] (XGB), and Gradient Boosting Machines [26] (GBM) and Elastic Net [27] (EN). These were selected for their ability to capture complex relationships and providing strong predictive performance and interpretability [28–30]. ERF, GBM and EN were implemented using the Python package scikit-learn (v1.7.2), and XGB used the Python package xgboost (v3.0.5).

Kolmogorov–Arnold Networks (KANs), based on the Kolmogorov–Arnold theorem, are a novel deep learning model [31, 32] that outperforms traditional Multi-Layer Perceptrons (MLPs) in interpretability [33, 34]. This theorem states that any multivariate continuous function on a bounded domain can be expressed as a finite composition of continuous univariate functions and additions. Unlike MLPs, which use fixed activation functions and linear weights, KANs employ learnable univariate functions (B-splines) along edges, enabling flexible modeling of complex, nonlinear relationships with smaller networks.

A defining feature of KANs is their intrinsically symbolic nature, which sets them apart from conventional machine learning models like Random Forests, GBMs, or MLPs. By representing activation functions as splines, KANs can be further symbolized into explicit mathematical expressions. This process, facilitated by grid extension and sparsity-inducing regularization, produces compact formulas that describe the model’s decision-making process. Unlike traditional models that often rely on post-hoc methods (e.g., SHapley Additive exPlanations [35] or Local Interpretable Model-agnostic Explanations [36]) for explanation or provide simple variable importance, KANs’ symbolic functions enable direct human understanding and visualization. For details on model development and application, see Chen et al. [37]. KAN was implemented using the Python package pykan (v0.2.8).

### Model training and evaluation metrics

All five models were deployed across *Epidemiology Dataset*, *Genetic Dataset*, and their *Combined Datasets*. A 5-fold cross-validation strategy with varying randomization was implemented to split the data into training and testing sets. Each model was finely tuned and trained on the training set, evaluated on the testing set, with predictions collected from all five testing folds for further analysis.

Model performance was assessed using the Coefficient of Determination (R²) score, with additional metrics, including Root Mean Square Error (RMSE), Mean Absolute Percentage Error (MAPE), and confusion matrices for BMI classification into four categories at ages 17, 20, 23, and 27). The RMSE, MAPE and confusion matrices are provided in Supplementary 2. The best-performing model and dataset were selected for further analysis and results presentation. KAN’s symbolic regression produced explicit mathematical formulas to describe its decision-making process, using activation functions selected for optimal performance. Previously identified key predictors, such as year 5 BMI z-score (*Y5BMIz*) and polygenic scores (*PGS002313, PGS002161, PGS000921*), were analyzed for their symbolic relationships with BMI outcomes [37], justifying their roles in predictions.

## Results

### Models and datasets comparison

Table 3 presents the R² values of five models trained on *Epidemiology Dataset*, *Genetic Dataset*, and their combination across the seven age groups. Among these models, the KAN models consistently outperformed the others across all age groups. When comparing the impact of datasets on BMI estimation, the combined use of *Epidemiology* and *Genetic Datasets* yielded the best results, particularly for age groups over 17 years, agreed by all the five models, followed by *Epidemiology Dataset* alone.

**Table 3:** Model performance (R^2^) across the seven age groups and the five models, by using Epidemiology and Genetic Datasets and the combination datasets.

Dataset	Model	Age (year)						
		8	10	14	17	20	23	27
Epidemiology	EN	0.78	0.61	0.51	0.38	0.37	0.34	0.24
	ERF	0.8	0.58	0.48	**0.42**	0.33	0.28	0.24
	GBM	0.79	0.6	**0.52**	0.36	0.35	0.26	0.23
	KAN	**0.81**	**0.62**	0.49	0.39	**0.38**	**0.37**	0.23
	XGB	0.78	0.61	0.46	0.39	0.35	0.27	0.21
Genetic	EN	0.12	0.13	0.16	0.1	0.13	0.12	0.14
	ERF	0.12	0.13	0.15	0.1	0.12	0.11	0.14
	GBM	0.12	0.13	0.12	0.07	0.09	0.09	0.11
	KAN	0.12	0.14	0.15	0.1	0.13	0.11	0.14
	XGB	0.11	0.12	0.12	0.07	0.1	0.09	0.11
Epidemiology and genetic	EN	0.78	0.64	0.52	0.41	**0.39**	0.34	0.29
	ERF	0.8	0.63	0.51	**0.44**	0.38	0.31	0.33
	GBM	**0.81**	0.63	0.46	0.39	0.38	0.35	0.31
	KAN	**0.81**	**0.65**	**0.55**	**0.44**	**0.39**	**0.38**	**0.34**
	KAN-f^a^	0.8	0.65	0.51	0.42	0.38	0.33	0.34
	XGB	0.79	0.62	0.48	0.41	0.35	0.33	0.28

The bold font indicates the highest R^2^ for the age.

^a^Symbolic formula result.

Regarding the influence of age, all models agreed that performance gradually decreased with age, ranging from R^2^ of 0.81 at the age of 8 years to 0.34 at the age of 27 years. Notably, the models after symbolic regression exhibited performance that closely aligned with their original counterparts, showing minimal differences.

As the KAN models trained on the combined datasets yielded the best results, the subsequent results and analyses will focus on the KAN models utilizing both datasets.

### Overall feature importance

The feature importance in the decision-making process of the KAN model was determined by multiplying the weights of each activation function from the leaf nodes (input variables) to the output (the BMI values of a target age group), with the visualized pruned tree plots shown in Supplementary 2.

**Fig. 1:**
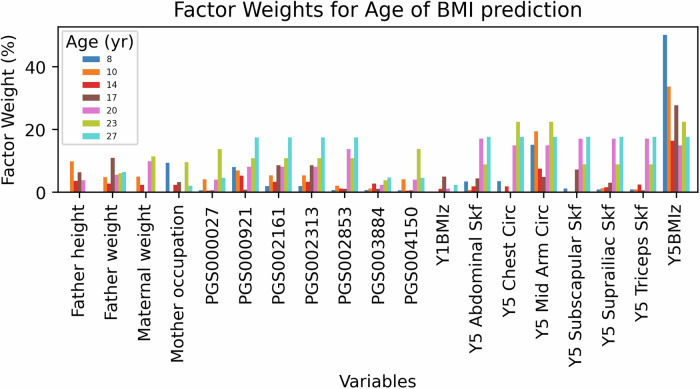
Key variables and their weights (in percentages) across seven age groups. Only variables appearing in at least four age groups are shown.

As shown in Fig. 1, the weights exhibit significant variation across different age groups and variables. Overall, the most influential variable is the BMI z-score at 5 years of age (*Y5BMIz*), with weights of 50%, 34%, 16%, 28%, 15%, 22% and 18% across the seven age groups, respectively. It demonstrates its dominance in early age groups. While its importance declines, it maintains similar absolute weight as several of the polygenic risk scores at the age of 27. In contrast, polygenic scores (PGS), including *PGS002313*, *PGS002161*, *PGS000027*, *PGS003884*, *PGS002853*, and *PGS000921*, have a lower impact in early age groups but gradually catch up with *Y5BMIz* after age 17.

Other anthropometry at age 5, including skinfolds (abdominal, suprailiac, subscapular and triceps), and arm and chest circumference, follow a similar trend in the models, with their weights increasing across the age groups. Variables consistently predicting offspring BMI across all age groups include maternal occupation, pre-pregnancy weight, late-pregnancy weight, maternal height-to-weight ratio, paternal weight, and paternal height. Additional variables, selected as important for specific ages not shown in Fig. 1, include pre-pregnancy and pregnancy smoking (ages 8, 10, 14), paternal occupation (ages 10, 14, 27), maternal age (ages 14, 17, 27) and childcare (ages 14, 17, 23). Interestingly, other factors that have been investigated in the literature for their association with subsequent offspring obesity [38–43] did not show influence in longitudinal models. This included factors such as delivery mode (age 14), placenta weight (ages 14), birth BMI z-score (age 17), solid food introduction age (age 17), paternal education (age 20), sex (age 20), breastfeeding (age 23), birth anthropometrics (age 23), Apgar score (age 23), maternal height (age 23), paternal age (age 27), and birth month (age 27). Further details are provided in Supplementary 1.

### Symbolic formulas

Our results demonstrate that the models maintain comparable performance after symbolic regression, as shown in Table 3 (“KAN-f*”). This consistency enables further exploration of mathematical relationships between key variables and their roles in predicting BMI. The symbolic formulas for BMI estimation, as a function of the selected variables across the seven age groups, are provided in Supplementary 1.

To identify and analyze the most influential factors across age groups, we examined the formulas associating *Y5BMIz* and the most heavily weighted polygenic scores *PGS002313* [15], *PGS002161* [16] and PGS000921 [21] along with predicted BMI values.

The BMI estimation and *Y5BMIz* formula across seven age groups are shown in Supplementary 2. *Y5BMIz*, a key factor among 10–20 in the KAN model’s decision-making, follows an exponential or first-quadrant sine function before age 20, mirroring actual BMI values and model estimations. After age 20, this relationship weakens due to data sparsity, with many data points in the lower range of *Y5BMIz* and true BMI, limiting the KAN model’s ability to derive a stable formula. However, the model uses additional variables to maintain predictive performance. These findings are further explored in Section “Role of early-life anthropometry and environmental factors”.

The influence of PGS on predicted BMI increases with age, with consistent positive correlations observed for *PGS002313*, *PGS002161*, and *PGS000921* across all age groups. The KAN model derived functional representations for the PGS pair (*PGS002313*, *PGS002161*) and *PGS000921*, shown in Supplementary 2 alongside BMI estimations. Most functions capture the positive correlation between these PGSs and BMI, except at age 17, where *PGS000921* has lower weight. We further discussed the impact of PGSs in Section “Impact of Polygenic scores (PGS)”.

## Discussion

### Key findings

This study explored over 200 variables, including maternal, early-childhood, familial, and genetic SNP data, to predict those at risk of future overweight and obesity, using KAN machine learning. Symbolic formulas from KAN address the “black box” issue, enabling transparent clinical decision-making with direct human visualization. Early-life factors like maternal weight during pregnancy, paternal height, and age 5 anthropometry strongly predict BMI in younger age groups. Their influence wanes in adolescence and early adulthood, where adult BMI polygenic scores (*PGS002313*, *PGS002161* and *PGS000921*) gain significance. Further, many early life factors which have been extensively investigated for their associations with subsequent offspring obesity, including birth weight [40] were not consistently present in these prediction models, suggesting that their limited role for offspring BMI prediction at a population level. These findings align with literature highlighting the balanced role of several dominant early-life conditions in shaping long-term BMI outcomes [44], which can be mitigated by further subsequent factors.

### Role of early-life anthropometry and environmental factors

Across the early preschool ages explored (birth, 1 and 5 years), anthropometry at age 5 years were selected by the models, particularly *Y5BMIz*, as the most influential predictor of subsequent BMI. *Y5BMIz* showed a persistent relationship with BMI in later life, underscoring its potential as a clinical biomarker for assessing the risk of adult overweight and obesity. Removing *Y5BMIz* from the full models, drastically reduces model effectiveness: R² drops from 0.81 to 0.70 at age 8, from 0.65 to 0.57 at age 10, and to near zero (0–0.01) for ages 14–27. This suggests that *Y5BMIz* captures early-life patterns that substantially drive longitudinal BMI tracking into adulthood. Without it, the model’s predictive ability for adolescence and adulthood BMI is largely lost. This finding is consistent with trajectory modeling of childhood obesity showing that, from age 5, obesity tracking is stable [45], while prior to this, there is cross over and catch up growth in some subsets. For example, some individuals exposed to adverse conditions in utero (e.g., malnutrition, smoking) may exhibit catch-up growth in those preschool years, which is associated with adult obesity risk [46], as outlined in the developmental origins of health and disease framework [47]. Further, early adiposity rebound (mean age 5.5 years) has been shown to be associated with adult obesity [48].

At older ages, skinfold thickness at age 5 surpasses *Y5BMIz* as a predictor, highlighting the value of body composition [49]. While some studies show BMI is as accurate as skinfolds for cardiovascular risk prediction [49–51], skinfolds better measure subcutaneous fat, which tracks in childhood [52]. This may reflect earlier subcutaneous fat saturation, leading to ectopic fat accumulation and metabolic dysfunction by the mid-twenties [53]. At a histopathological level, subcutaneous tissue in overweight children had greater adipocyte surface area and collagen content in their subcutaneous tissue compared to normal weight children [54].

Despite including many early-life variables (such as preschool exposures), none were selected in final KAN models, suggesting they may not reliably predict population-level obesity risk.

### Impact of polygenic scores (PGS)

Polygenic scores (PGS) enhanced BMI prediction only when combined with epidemiological factors. In particular, three of the seven PGSs, *PGS002313* [15], *PGS002161* [16] and PGS000921 [21] were top predictors across multiple ages, showing consistent positive correlations with BMI (Supplementary 2). *PGS002313* and *PGS002161*, both developed in the UK Biobank (BOLT-LMM and LDpred2, respectively), are strongly correlated (*r* > 0.9) and in turn, moderately correlated with *PGS000921* (*r* > 0.6, Inter99 cohort, LDpred), suggesting shared SNP signals critical for adult BMI. In contrast, *PGS000027* and *PGS004150*, also from UK Biobank, are correlated but less predictive, likely capturing less informative variation.

For the three most informative PGSs, the KAN model revealed non-linear relationships, such as exponential-like functions for *PGS002313* and *PGS002161* at ages 14, 17, and 20. (Supplementary 2).

The influence of PGSs tested in this study increased from childhood to adulthood. Notably, the PGSs were originally developed from adult cohorts, which may explain their stronger predictive performance for BMI in adulthood compared to childhood. Further, variability in BMI in childhood may need to be captured by different PGSs due to varying genetic influences across life stages [55–57]. To explore this, we assessed pediatric PGSs (*PGS000716* [58], *PGS004610* [59], *PGS004900* [60]) from the UK Biobank, based on self-reported body size at age 10. However, they were excluded due to potential recall bias and imprecision.

We concur with evidence from previous studies [55, 56], which indicate that adult BMI PGSs can provide meaningful predictive power for adolescent BMI, indicating some genetic overlap across ages. Nevertheless, we suggest that there is a need for PGS developed specifically for childhood and adolescence BMI, where a slightly different set of influences are at play.

### Influence of parental factors

Parental factors play a pivotal role in BMI outcomes, with variables such as parental education, household income, parental height and weight, maternal age at childbirth, and maternal weight gain during pregnancy showing consistent associations across all age groups. These factors likely capture both genetic and environmental influences. For instance, parental education and household income reflect socioeconomic and environmental conditions that influence health behaviors and resource access. Similarly, maternal weight gain during pregnancy may affect fetal development through nutritional and metabolic pathways. These associations highlight the need for a holistic framework integrating genetic, maternal, and environmental elements in BMI prediction models.

The influence of these factors evolves with age: maternal and paternal anthropometric measures have limited impact in early childhood but grow stronger in adolescence and early adulthood, aligning with the rising role of adult BMI PGSs in later stages.

### Strengths and limitations

Overall, the strength of this study lies in integrating nearly three decades of prospectively collected genetic and epidemiological data using KAN machine learning, which can both predict and simultaneously provide mathematical transparency in predicting adult BMI utilizing information that is available at 5 years old and before.

However, there are also several limitations to this study, namely using BMI as a surrogate measure of adiposity, the fixed time points of data collection, inability to create sex-specific models, and inability to imply causation. Using BMI as the sole obesity indicator may not fully capture adiposity complexity. Alternative measures (e.g., waist circumference, waist-to-hip ratio) could complement BMI. A further limitation is the use of fixed time points for anthropometric data collection (birth, 1 year, and 5 years), preventing assessment of other time points. Individual risk, however, may be better captured by BMI velocity with repeated measures with other time points.

We opted to develop unified models across sexes, as sex was a significant predictor only at age 20 with lower weightings than *Y5BMIz* or PGSs. Further, childhood and adolescent BMI z-scores already account for sex-related growth differences. However, sex-specific models may reveal further insights in larger sample sizes where the halving the sample size, limiting statistical power were not an issue. Finally, no causation is implied in this study, as selected variables may capture latent variance rather than direct effects.

While we categorized data into *epidemiological* and *genetic* datasets, these are not entirely distinct. Anthropometric measures like *Y5BMIz*, parental height, and weight likely reflect genetic influences due to heritability. This design effectively models combined effects but is less suited for separating genetic and environmental contributions, unlike the twin studies [61], which show increasing BMI heritability from infancy to adulthood, consistent with our finding that PGS influence strengthens post-adolescence while early-life factors dominate in childhood.

The KAN model slightly outperformed other machine learning models in R² scores, but is computationally complex compared to simpler models like Elastic Net. Its strength lies in providing symbolic formulas that reveal variable relationships, feature importance, and effect directions (Supplementary 2). However, the symbolic regression attribute of KANs struggled with weak relationships or low feature weights, such as *PGS000921* at age 17, *PGS002313* and *PGS002161* at age 8, and *Y5BMIz* and BMI at ages 20 and 23 (Supplementary 2, Supplementary Section 5). Despite these challenges, KAN’s interpretable insights make it valuable for avoiding the “black box” interpretation of machine learning and allowing transparency when using the information to drive clinical decision making. Its use of information that is available at 5 years old and before makes it suitable for application to early intervention of childhood and prevention of adult obesity.

### Future directions and conclusion

The findings underscore the importance of aggregating the combined effects of early-life factors, fixed genetic variants, parental and maternal influences in shaping BMI trajectories from childhood to early adulthood. These insights pave the way for more targeted and effective interventions aimed at promoting healthy weight and preventing obesity over the life course.

Future studies should follow up these findings to ascertain if different fixed genetic variants can contribute patterns of childhood growth and whether skinfold thickness or more sophisticated measures of body composition (subcutaneous fat thickness measurements) has increased importance.

## Supplementary information

Supplementary summary

Supplementary Material 1

Supplementary Material 2

## Data Availability

The datasets generated during and/or analyzed during the current study are not available. The Raine study is committed to a high level of confidentiality of the data in line with the informed consent provided by participants. Requests for data should be directed to the Raine Study Executive.

## Footnotes

1 The variables in the Rain Study are mostly harmonized with the LifeCycle Project-EU Child Cohort Network [14].

## Code availability

Machine learning model coding is available upon reasonable request.
