# Supplementary material for: Longitudinal prediction of BMI using explainable AI: integrating polygenic scores, maternal, early-life and familial factors
Source: Int J Obes (Lond). 2026 Mar 16;50(5):1142–9. doi: 10.1038/s41366-026-02050-1 (PMC13226034; doi:10.1038/s41366-026-02050-1)
Supplement: Supplementary file 1 — Supplementary summary [file 41366_2026_2050_MOESM1_ESM.docx]

Supplementary 1.xlsx file contains

- Sheet 1 – All variables: the names, descriptions, values, and the metadata of all the variables used in this study.
- Sheet 2 – Key characteristics statistics: the summary statistics of some key cohort characteristics.
- Sheet 3-9 – yr8/10/14/17/20/23/27 KAN weights: selected features, inclusive raw variables and the weights of the features generated by the models to estimate year 8, 10, 14, 17, 20, 23, 27 BMI.
- Sheet 10 – Symbolic regression: The standardised formulas of the BMI calculation derived from the age-specific models.

Supplementary 2.pdf file contains

1. Data Processing
   The dataset preprocessing workflow
2. Algorithm of variables clustering and selection
   It describes the method used for high-correlated variables clustering, and the data size of each age group before and after clustering
3. Model performance
   Depart from the model performance of R^2^ presented in the manuscript, it shows additional metrics, including
   3.1 Root Mean Squared Error,
   3.2 Mean Absolute Percentage Error,
   3.3 Confusion matrix for age 17, 20, 23 and 27 years
4. Pruned tree plots of the KAN models
   The pruned tree plots after training the KAN models for age 8, 10, 14, 17, 20, 23 and 27 years.
5. BMI estimation and the expression of the key variables
   It contains two sets of plots that show the models formularization on Y5BMIz and PGS7 across the seven age groups.
